# Numerical Simulations of MitraClip Placement: Clinical Implications

**DOI:** 10.1038/s41598-019-52342-y

**Published:** 2019-11-01

**Authors:** Ramji Kamakoti, Yaghoub Dabiri, Dee Dee Wang, Julius Guccione, Ghassan S. Kassab

**Affiliations:** 1 Dassault Systémes Simulia Corporation, Minneapolis, MN USA; 2 Center for Structural Heart Disease, Henry Ford Health System, Detroit, MI USA; 3 UCSF Department of Surgery, San Francisco, USA; 4 California Medical Innovations Institute, San Diego, CA USA

**Keywords:** Biomedical engineering, Cardiac device therapy

## Abstract

Mitral regurgitation (MR) is the most common type of valvular heart disease in patients over the age of 75 in the US. Despite the prevalence of mitral regurgitation in the elderly population, however, almost half of patients identified with moderate-severe MR are turned down for traditional open heart surgery due to frailty and other existing co-morbidities. MitraClip (MC) is a recent percutaneous approach to treat mitral regurgitation by placement of MC in the center of the mitral valve to reduce MR. There are currently no computational simulations to elucidate the role of MC on both the fluid and solid mechanics of the mitral valve. Here, we use the Smoothed Particle Hydrodynamics (SPH) approach to study various positional placements of the MC in the mitral valve and its impact on reducing MR. SPH is a particle based (meshless) approach that handles flow through narrow regions quite efficiently. Fluid and surrounding anatomical structure interactions is handled via contact and hence can be used for studying fluid-structure interaction problems such as blood flow with surrounding tissues/structure. This method is available as part of the Abaqus/Explicit solver. Regurgitation was initiated by removing targeted chordae tendineae that are attached to specified leaflets of the mitral valve and, subsequently, MC implants are placed in various locations, starting from the region near where the chordae tendineae were removed and moving away from the location towards the center of the valve. The MC implant location closest to where the chordae tendineae were removed showed the least amount of residual MR post-clip implantation amongst all other locations of MC implant considered. These findings have important implications for strategic placement of the MC depending on the etiology of MR to optimize clinical outcome.

## Introduction

Heart failure (HF) is a worldwide epidemic with over 6 million afflicted patients and 600,000 new patients diagnosed each year that contributes considerably to the overall cost of health care in developed nations. The number of people afflicted with this complex disease is increasing at an alarming pace; a trend that is likely to continue for many years to come. Mitral regurgitation (MR) is the most common type of valvular heart disease in patients over the age of 75 in the US^1^; a condition that exacerbates HF. In the US alone, there is an estimated 4 million patients with moderate-severe mitral regurgitation, MR (i.e., 3+ or 4+). However, despite the prevalence of mitral regurgitation in the elderly population, almost half of patients identified with moderate-severe MR are turned down for traditional open heart surgery due to frailty and other existing co-morbidities^1^. MitraClip (MC) is a recent percutaneous approach to treat mitral regurgitation by placement of MC in the center of the mitral valve to reduce MR. Until recently, surgical repair of the mitral valve has been used to treat MR, but over half of the patients with severe MR are not indicated for surgery because of age, comorbidities, and/or impaired left ventricular (LV) function. A less invasive approach for MR treatment is required for a large number of these patients which has given rise to a minimally invasive catheter-delivered clip (MitraClip or MC) for the treatment of MR (approved in Europe/USA).

The MC device limits MR by replicating the Alfieri procedure, in which mitral valve leaflets are connected at a point that facilitates improved coaptation, creating a dual orifice valve. The clip is deployed via a catheter under fluoroscopic and echocardiographic guidance by crossing the atrial septum and advancing from the left atrium into the LV just below the mitral valve. Once deployed, the MC keeps the region of the valve responsible for the MR closed, while the adjacent valve regions are able to open and close to allow the flow of blood. Improvements in HF class and reduced mortality and hospitalizations are consistently reported in the literature after MC placement^2^. Additionally, cost utility analysis found results were robust to changes in all clinical parameters. The MC device is FDA approved and represents an important therapy for MR patients deemed too sick for surgery, but also as an alternative to more invasive cardiac surgery treatment for the remaining MR patient population.

Incorrect placement or poor positioning of the MC (e.g., off center, too shallow, too deep, etc.) has implications for performance of the device and hence therapy including persistent MR, clip slippage or erosion, need for multiple clips (higher expense), etc. Anatomical limitations to sufficient leaflet grasp by the clip, persistent left ventricular remodeling and progression of underlying mitral annular dilation post MC implantation are all risk factors associated with recurrent mitral regurgitation despite percutaneous repair. Recurrent mitral regurgitation of at least moderate severity is clinically associated with adverse left heart dilation, heart failure readmission, and increased risk of death^3^. Currently, there is lack of understanding of optimal placement of the MC and associated cardiac and LV flow implications. To our knowledge, there is currently no computational simulation of the flow in conjunction with MC implant nor the distribution of stresses imposed by the clip on the mitral leaflets. Hence, the goal of this paper is to introduce a fully coupled fluid structure interaction (FSI) model to understand the MC device flow and stress interactions to optimize therapy.

## Methods

The underlying methodology used to simulate fluid (blood) flow is based on smoothed particle hydrodynamics (SPH) in Abaqus (Abaqus 6.14 Analysis User’s Guide, Section 15.2). This methodology is a numerical method that is part of the larger family of meshless (or mesh-free) methods^4^. For these methods, it is not necessary to define nodes and elements normally done in a finite element (FE) analysis. Instead, only a collection of points is necessary to represent a given body. In SPH, the nodes are commonly referred to as particles or pseudo-particles. SPH addresses modeling needs in cases where traditional mesh-based methods (FE, finite difference) fail or are inefficient, such as violent fluid flows, extremely high deformations/obliterations, pinched flow, flow through narrow orifices, etc.^5^. The novelty of SPH is related to interpolation and differentiation between particles^5,6^, and the velocity of each particle is dependent on all its neighbor particles (Abaqus 6.14 Analysis User’s Guide, Section 15.2).

SPH analysis is an Abaqus/Explicit capability implemented for 3-D models. Any of the material models available in Abaqus/Explicit can be used including user-defined materials. Initial conditions, boundary conditions and loads can be specified as for any Lagrangian model.

### Finite element to particle conversion

One of the key features of SPH in Abaqus is the ability to convert underlying FE meshes into particles. The power of both Lagrangian FE and SPH methods can be brought together when solving a complicated mechanics problem. The FE method can be used to model the initial stages of the deformation and then, when the mesh distortions become significant, they can be converted to SPH particles. During simulation, newly activated particles are in interaction with dormant (not activated) and already-activated particles. Number of particles in each element can be specified, but it also depends on element type (Abaqus 6.14 Analysis User’s Guide, Section 15.2).

#### Computational setup

**Figure 1:**
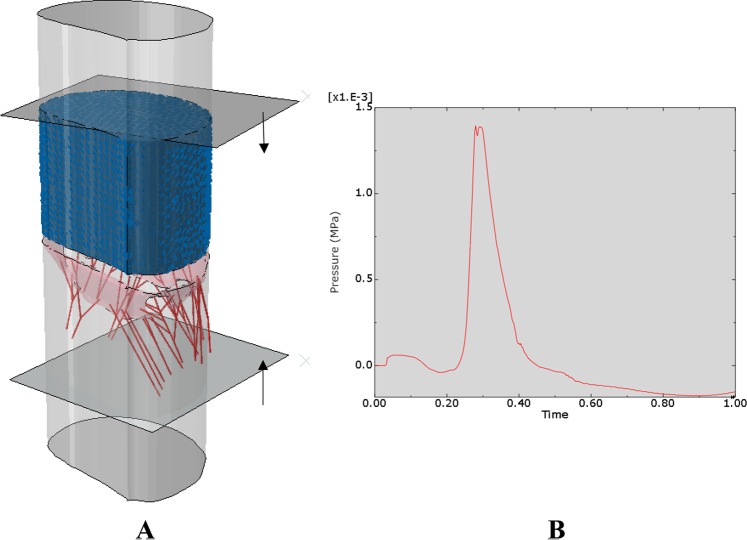
(**A**) Computational model setup including mitral valve, chordae and SPH particles representing blood flow. (**B**) Pressure load applied to the valve. These figure were obtained in Abaqus 6.14, https://www.3ds.com/products-services/simulia/products/abaqus/.

The computational model utilized in this study is a multi-physics *Abaqus/Explicit* FE simulation^7^ combining both fluid and structural dynamics (Fig. 1). The fluid portion of the simulation utilizes SPH technique with a total of 31,000 particle elements representing the fluid region. The underlying valve geometry, which represents the solid portion of the model, is adapted from the three-dimensional computer-aided design model from^8^. The valve is modeled using an anisotropic hyper-elastic material model^9^ and is meshed with 17,000 tetrahedral elements. The flexible tube that is used to represent the heart chambers (Left Atrium and LV) is modeled as surface elements with contraction and expansion. The MC was implemented using a combination of distributed coupling and connector elements to bring the leaflets together to mimic the MC device (the actual device itself was not implemented). The top plate is moved downwards to mimic the ejection cycle of the mitral valve while the bottom plate is moved upwards to mimic the closing cycle (testing MR). During this cycle, the mitral valve and tube are subject to a time varying pressure boundary condition as depicted in Fig. 1 to simulate the expansion/contraction of the mitral valve. Total computational time for one analysis (open/close cycle of the valve) is about 4 hours using 36 CPUs.

**Figure 2:**
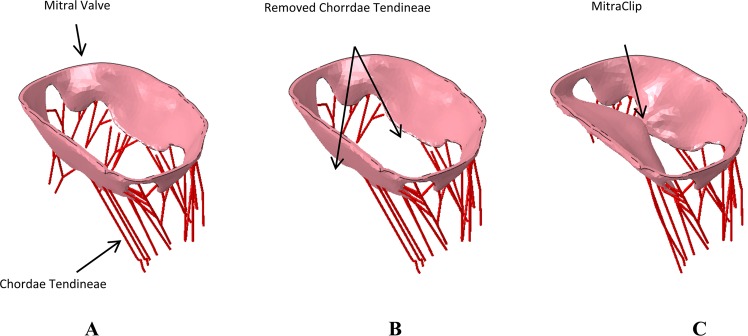
(**A**) Normal mitral valve. (**B**) Diseased mitral valve with some of the chordae removed. (**C**) Mitral valve with MitraClip implant. These figure were obtained in Abaqus 6.14, https://www.3ds.com/products-services/simulia/products/abaqus/.

**Figure 3:**
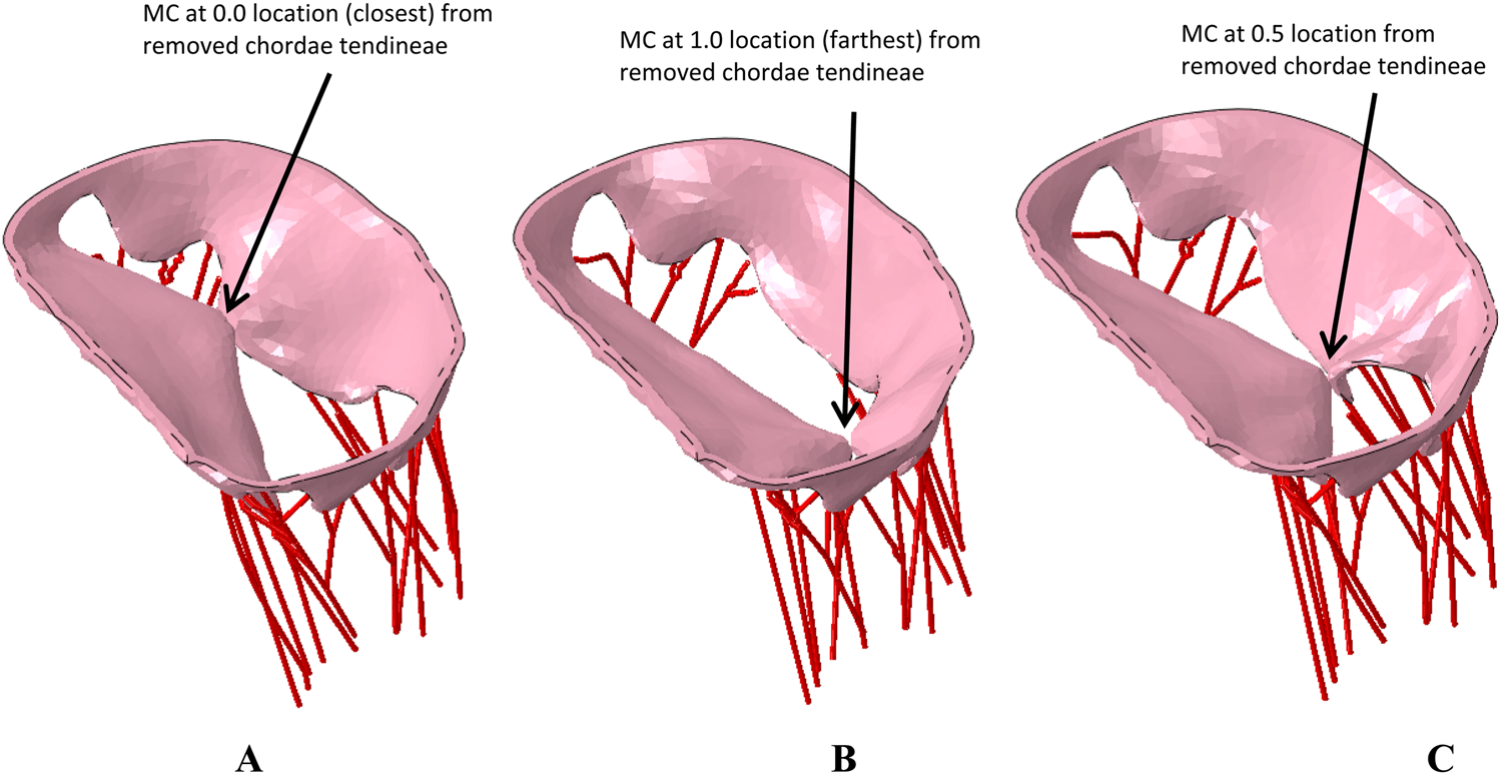
(**A**) Mitral valve with MC implant at the location of missing chordae location. (**B**) Mitral valve with MC implant a little offset from missing chordae location. (**C**) Mitral valve with MC implant further offset from missing chordae location. These figure were obtained in Abaqus 6.14, https://www.3ds.com/products-services/simulia/products/abaqus/.

Multiple scenarios were analyzed to understand the potential efficacy of the MC device in (a) Normal mitral valve, (b) Diseased mitral valve apparatus (missing chordae tendineae), and (c) Diseased mitral valve treated with the MC implant (Fig. 2) in various locations of the mitral valve (Fig. 3). We considered three placement locations of the MC based on the distance from the valve leaflets where the chordae tendineae were removed (where 0 denotes the location closest to or at the location of where the chordae tendineae were removed and 1 is the furthest distance from the leaflets where the chordae tendineae were removed). Based on this, the 3 locations of the MC implant considered were: (1) Zero location; (2) 1.0 location or the other end of the leaflets from where the chordae tendineae were removed; and (3) 0.5 location or half-way between the other end of leaflets and where the chordae tendineae were removed (demonstrated in Fig. 3). The role of various locations of the MC implant on regurgitation fraction and leaflet stress distribution was considered.

## Results

Numerical results were produced using the SPH methodology to evaluate both the response of a compromised mitral valve, as well as the efficacy of the implantation of MC. Three situations were analyzed for the comparison (1): Normal mitral valve (2), Compromised mitral valve, and (3) Compromised valve treated with MC. The initial configuration of each of these models are shown in Fig. 2.

**Figure 4:**
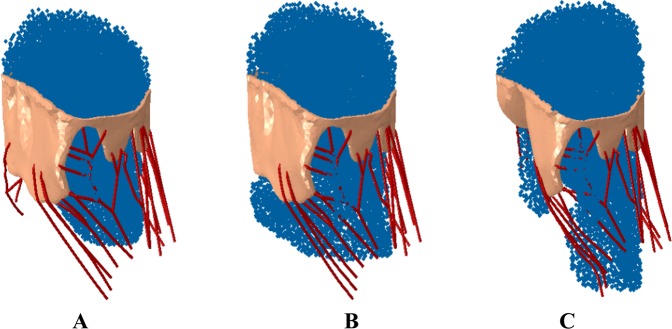
Fluid flow through the mitral valve in (**A**) Normal. (**B**) Compromised chordae tendineae. (**C**) Treated with MC during full opening of the valve. These figure were obtained in Abaqus 6.14, https://www.3ds.com/products-services/simulia/products/abaqus/.

**Figure 5:**
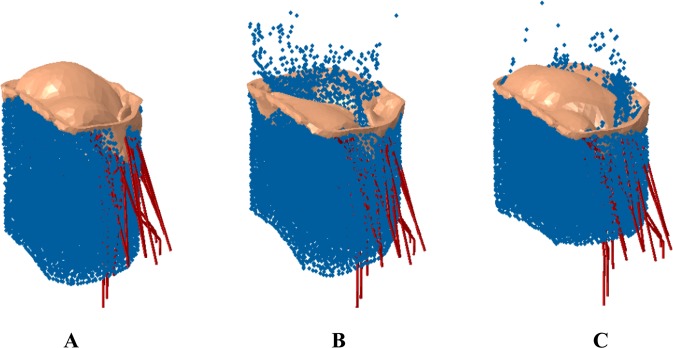
(**A**) Normal mitral valve exhibiting no leakage. (**B**) Diseased mitral valve showing significant flow back into left atrium. (**C**) Mitral valve with MC implant exhibiting reduced leakage. These figure were obtained in Abaqus 6.14, https://www.3ds.com/products-services/simulia/products/abaqus/.

The simulation begins with fluid flowing through the valve (LV filling), and then valve closure during the ejection phase of the cardiac cycle as shown in Fig. 4. The tendency of any fluid to flow through the mitral valve during the ejection phase is observed in the three situations outlined above to assess mitral valve regurgitation. As can be observed from Fig. 5, no MR is observed for the normal simulation case, significant MR is observed for the simulation with compromised chordae tendineae, and reduced MR for the mitral valve apparatus treated with the MC.

**Figure 6:**
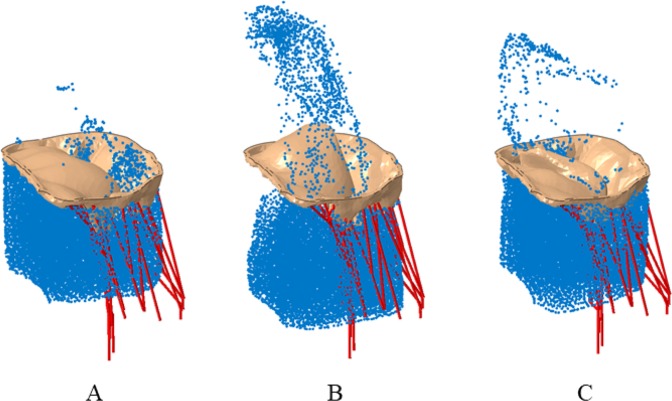
(**A**) Regurgitation of mitral valve with MC implant close to missing chordae showing minimal regurgitation. (**B**) Regurgitation of mitral valve with MC implant offset to missing chordae showing some regurgitation. (**C**) Regurgitation of mitral valve with MC implant offset farthest from missing chordae showing more regurgitation. These figure were obtained in Abaqus 6.14, https://www.3ds.com/products-services/simulia/products/abaqus/.

**Figure 7:**
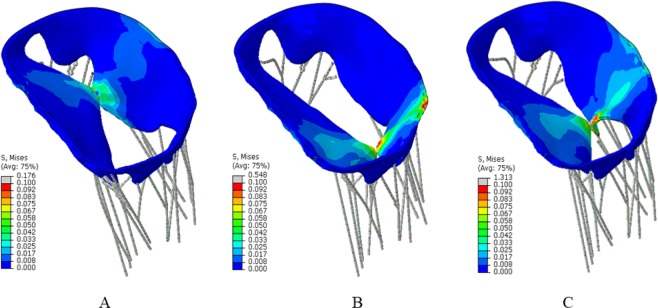
Stresses on the mitral valve for the different MC implant placements. (**A**) MC implant close to missing chordae, (**B**) MC implant offset to missing chordae, (**C**) MC implant offset farthest from missing chordae. These figure were obtained in Abaqus 6.14, https://www.3ds.com/products-services/simulia/products/abaqus/.

Figure 6 shows the predicted MR for the different scenarios described above; (1) 0 location or near the chordae, approximately one third across the mitral valve; (2) 1.0 location or roughly three-fourth across the mitral valve and (3) 0.5 location or roughly halfway across the mitral valve. The regurgitation fraction (defined as amount of fluid regurgitated vs amount in chamber prior to ejection phase) for the 3 different MC implant locations were approximately 3.5%, 4% and 10%, for the 0, 0.5, and 1.0 locations, respectively. Specifically, the greatest amount of regurgitation was observed for the no MC case, followed by the one with MC placed farthest from the missing chordae tendineae location and the MR was significantly improved when the MC implant was placed close to the missing chordae tendineae location (shown in Fig. 6). The stresses experienced during the MC implant process in the mitral valves for the various placement locations are shown in Fig. 7.

## Discussion

There are currently no validated mathematical models that describe the MC device and the associated effect on fluid or solid mechanics of the mitral annulus and leaflets. Here, we introduce a fully coupled FSI model of the MC device. The metric to gauge the efficacy of the MC implant in reducing MR is the tendency of reverse flow (i.e., flow from the LV to the left atria) during the LV ejection phase of the cardiac cycle.

There are a number of potential issues with incorrect placement of MC including (1): No efficacy (i.e., no reduction of insufficiency; e.g., either off center MC placement or single leaflet attachment) (2), Damage of leaflets or MC entangled in cords making repair at a later date very difficult (3), Perforation of the leaflet, and (4) Creation of mitral stenosis. Some of these complications are due in part to incorrect transseptal (TS) entry point. A very specific TS access point on the superior and posterior portion of the *fosa ovalis (*FO) is required for MC procedures. If access at this location is not achieved, some of the complications noted above may occur due to less than optimal positioning/delivery. Additionally, significant procedural and x-ray exposure time will be added as unsuccessful attempts will be made to place the MC, the MC catheter will be removed, TS access will be reestablished, and the MC catheter will be re-advanced. The consequences of less than optimal placement can have hemodynamic implications as investigated in this study.

Therefore, an efficient MC implantation depends on several factors including the procedural success and the procedure time^10,11^. Procedural failure could be a high risk for MC therapy particularly for patients with frailty^10–12^. Our computational framework could guide the interventional cardiologist to avoid procedural failure and to implant MC in a shorter time. Other than the location of MC, other factors such as number of MCs can be planned prior to the interventional procedure using our computational framework. Key parameters such as stresses within the leaflets (Fig. 7) and post-procedural regurgitation (Figs 5 and 6) can be simulated, leading to a more efficient MC therapy.

Although this study is in its early feasibility and infancy of development, the concept of predicting patient outcomes based on personalized anatomical fluid-simulation modeling is novel to percutaneous MC therapy. With the ability to model patient specific anatomy and anticipated fluid or MR response to clip implantation, clinicians will have more optimal toolkits to utilize in patient candidacy determination for transcatheter therapies. As the transcatheter mitral arena broadens with newer valve devices, bands, and annular solutions, the ability to simulate for patient-specific device based therapy response will be critical to matching patients with the ideal type of transcatheter mitral intervention.

In our predictive modeling, there is a noted correlation between pathology and MC site of intervention. The numerical results predict no MR for the normal mitral valve as expected. The diseased mitral valve case exhibits MR, and the valve treated with the MC implant exhibits a reduction in the amount of MR. All cases performed satisfactorily when the valve was fully open (Fig. 4A–C) but there were some differences observed with different MC implant locations during the closing cycle, as pointed out below.

First, the normal mitral valve (Fig. 2A) completely closes, as expected, leading to virtually no regurgitation (Fig. 5A) during the ejection cycle. Second, the diseased mitral valve (Fig. 2B), where some chordae tendineae have been removed, exhibits significant regurgitation (Fig. 5B) as the valves are unable to close completely during the ejection cycle. Third, the regurgitation was significantly reduced (Figs 5C and 6) with the MC implant (Figs 2C and 3). Three locations were considered for the MC placement and depending on the placement, the regurgitation fraction varied; i.e., the one closest to the location of missing chordae tendineae (zero location, Fig. 3A) resulted in the least amount of MR (Fig. 6A) and the one farthest from the missing chordae tendineae (1.0 location, Fig. 3B) resulted in the greatest amount of MR (but less than the untreated case; i.e., no MC, Fig. 6B). The reason for these findings is as follows. First, for the case where the MC implant is at zeroth location (Fig. 3A), it plays the role of the missing chordae tendineae and hence makes up for the stiffness provided by the chordae tendineae during the opening/closing stages of the valve. Second, on the other hand, for the 1.0 location where the MC implant location is farther away from the missing chordae tendineae (Fig. 3B), the leaflets near the missing tendineae section hang loose and hence are unable to fully close during the cardiac cycle leading to more regurgitation (Fig. 6B). Third, for the 0.5 location (midway between the zero and 1.0 location, Fig. 3C), the leaflets still did not have the required stiffness from the missing chordae tendinea and hence some regurgitation (Fig. 6C) was still observed but not quite as much as 1.0 location or the missing MC case.

The Von-Mises stresses experienced (Fig. 7) by the valve leaflets for the various placements of the MC implant also varied depending on the location. The stresses were smallest for the ideal location of the MC (zero location, Fig. 7A) as the chordae tendineae do not pull the leaflets back when the MC is placed. The stresses do increase on the leaflets/wall of the mitral valve as the MC is placed further away (Fig. 7B,C) from the missing chordae tendineae location, however, as the tendineae resist the closing of the leaflets during the MC placement. There is roughly 10 times more stress experienced on the walls of the mitral valve for the 1.0 MC implant location and about 2 times more stress for the 0.5 case.

According to the best of our knowledge, we have assessed the regurgitation of MV after MC implantation, for the first time. The FSI has been reported for the MV in normal, diseased and treated conditions^13,14^, but MV regurgitation in MC therapy has not been studied using a FE approach. Because the regurgitation after MC implantation is a key parameter, our study provides important insights optimizing MC therapy for MV regurgitation.

This study is not without limitations. A simplified geometry, such as a flexible tube instead of the atrium/ventricles, was used to study the efficacy of the MC implant on MR. Furthermore, the MC implant was implemented via a combination of distributed coupling and connector elements on the leaflets instead of using an actual MC device. Finally, pressure-based boundary conditions were used on the mitral valve to mimic the actual loading experienced by the mitral valve during the cardiac cycle. A future development would be to perform the study using the Living Heart Human Model^15^ by placing the MC/mitral valve inside the beating human heart and observe the effects of MR with patient-specific boundary conditions. Despite the limitations, the present model provides significant insights into a current HF therapy which has not been previously simulated.

We did not consider the interaction between the leaflets and blood flow during a cardiac cycle. The opening and closing of the leaflets is a result of this interaction. However, this limitation does not change our findings because our study concentrated on regurgitation of the MV (that is related to the closed configuration of MV). A more comprehensive analysis would consider a fully coupled FSI during a cardiac cycle to study the leaflets dynamics and blood flow through the MV after MC.

The simulation results suggest the location of MC placement is important for both the degree of MR reduction as well as the stresses acting on the mitral leaflets. Ideally, patient specific models should be used to virtually plan placement of the MC for maximum reduction of MR and minimum increase of leaflet stresses. These optimizations would ensure long term safety and efficacy of the MC procedure for treatment of HF.
